# Desmoglein 2 promotes vasculogenic mimicry in melanoma and is associated with poor clinical outcome

**DOI:** 10.18632/oncotarget.10216

**Published:** 2016-06-22

**Authors:** Lih Yin Tan, Chris Mintoff, M. Zahied Johan, Brenton W. Ebert, Clare Fedele, You Fang Zhang, Pacman Szeto, Karen E. Sheppard, Grant A. McArthur, Erwin Foster-Smith, Andrew Ruszkiewicz, Michael P. Brown, Claudine S. Bonder, Mark Shackleton, Lisa M. Ebert

**Affiliations:** ^1^ Centre for Cancer Biology, University of South Australia and SA Pathology, Adelaide, SA, Australia; ^2^ School of Pharmacy and Medical Sciences, University of South Australia, Adelaide, SA, Australia; ^3^ Cancer Development and Treatment Laboratory, Peter MacCallum Cancer Centre, Melbourne, VIC, Australia; ^4^ Molecular Oncology Laboratory, Peter MacCallum Cancer Centre, Melbourne, VIC, Australia; ^5^ Sir Peter MacCallum Department of Oncology and Department of Pathology, University of Melbourne, VIC, Australia; ^6^ Division of Anatomical Pathology, SA Pathology, Adelaide, SA, Australia; ^7^ Cancer Clinical Trials Unit, Royal Adelaide Hospital, Adelaide, SA, Australia; ^8^ Discipline of Medicine, University of Adelaide, Adelaide, SA, Australia

**Keywords:** melanoma, desmoglein 2, vasculogenic mimicry, cadherin, TCGA

## Abstract

Tumors can develop a blood supply not only by promoting angiogenesis but also by forming vessel-like structures directly from tumor cells, known as vasculogenic mimicry (VM). Understanding mechanisms that regulate VM is important, as these might be exploitable to inhibit tumor progression. Here, we reveal the adhesion molecule desmoglein 2 (DSG2) as a novel mediator of VM in melanoma. Analysis of patient-derived melanoma cell lines and tumor tissues, and interrogation of The Cancer Genome Atlas (TCGA) data, revealed that DSG2 is frequently overexpressed in primary and metastatic melanomas compared to normal melanocytes. Notably, this overexpression was associated with poor clinical outcome. DSG2^+^ melanoma cells self-organized into tube-like structures on Matrigel, indicative of VM activity, which was inhibited by *DSG2* knockdown or treatment with a DSG2-blocking peptide. Mechanistic studies revealed that DSG2 regulates adhesion and cell-cell interactions during tube formation, but does not control melanoma cell viability, proliferation or motility. Finally, analysis of patient tumors revealed a correlation between DSG2 expression, VM network density and expression of VM-associated genes. These studies identify DSG2 as a key regulator of VM activity in human melanoma and suggest this molecule might be therapeutically targeted to reduce tumor blood supply and metastatic spread.

## INTRODUCTION

Without access to a blood supply, solid tumors cannot grow more than a few millimeters in diameter [1]. For many years, it was thought that the growth of vascular networks within tumors occurred exclusively via the sprouting and extension of pre-existing blood vessel networks into tumors via angiogenesis. Indeed, targeting this process in the clinic has produced life-prolonging therapies for a variety of cancers. However, the benefits obtained from current anti-angiogenesis therapies are modest and many cancer types, such as melanoma, respond poorly [2]. Thus, blocking classical angiogenesis may be only part of the solution to prevent malignant tumors from developing a blood supply.

In this context, much attention has recently focused on vasculogenic mimicry (VM), the formation of vascular networks directly by tumor cells [3, 4]. In VM, hollow channels and narrow conduits within tumors are lined with basement membrane, similarly to traditional blood vessels. However, in contrast to traditional vessels, VM channels are lined by tumor cells rather than endothelial cells (EC). The biological importance of VM to tumors is becoming increasingly clear, with several studies showing that VM channels can anastomose (fuse) with conventional blood vessels to access the blood supply, and possess lumens through which blood actively flows [5–9]. Furthermore, the presence of histologically-detected VM networks in primary tumors is predictive of poor survival and increased metastasis in patients with melanoma [10–14] and other cancers [3, 4, 15]. Similarly, in animal models, inhibiting VM in solid cancers leads to improved survival [16, 17], and a recent study found that breast cancer clones with the highest capacity to enter the vasculature and establish metastasis were those most efficient at generating VM networks [18]. Together, these findings highlight the biological and clinical significance of VM. Despite this, the molecular mechanisms that regulate VM are not well understood, although pathways associated with vasculogenesis, embryogenesis and hypoxia have been implicated [3, 4].

Desmoglein 2 (DSG2) is a surface-expressed adhesion molecule belonging to the cadherin family, and has a well-described function in the formation of desmosomes, a specific type of cell-cell junction found uniquely in epithelia and myocardium [19]. In humans, four desmoglein isoforms (DSG1-4) and three desmocollin isoforms (DSC1-3) have been identified, all of which undergo homotypic and heterotypic interactions to form the adhesive interface of desmosomes. Surprisingly, while melanoma cells are not thought to generate desmosomes, expression of DSG2 in this cell type has been described [20–22], though another study failed to detect DSG2 expression in human melanoma biopsies [23]. The biological function of DSG2 in melanoma remains poorly defined, although one study has linked its expression to the regulation of melanoma cell motility [21].

We recently identified a novel role for DSG2 in the endothelial cell lineage (Ebert *et al*, submitted). These studies demonstrated non-desmosome-localized expression of DSG2 by ECs and circulating endothelial progenitor cells (EPCs), and established a critical role for DSG2 in regulating the angiogenic activity of these cells. These findings, together with those of others [24], suggest an important role for DSG2 in regulating classical angiogenesis. Here, we reveal that DSG2 plays an analogous, cell-intrinsic role in melanoma by regulating VM. These findings suggest that therapeutic targeting of DSG2 may allow simultaneous inhibition of both classical angiogenesis and VM, and thereby provide a powerful new treatment approach for solid tumors such as melanoma.

## RESULTS

### DSG2 is heterogeneously expressed by melanoma cell lines

**Figure 1:**
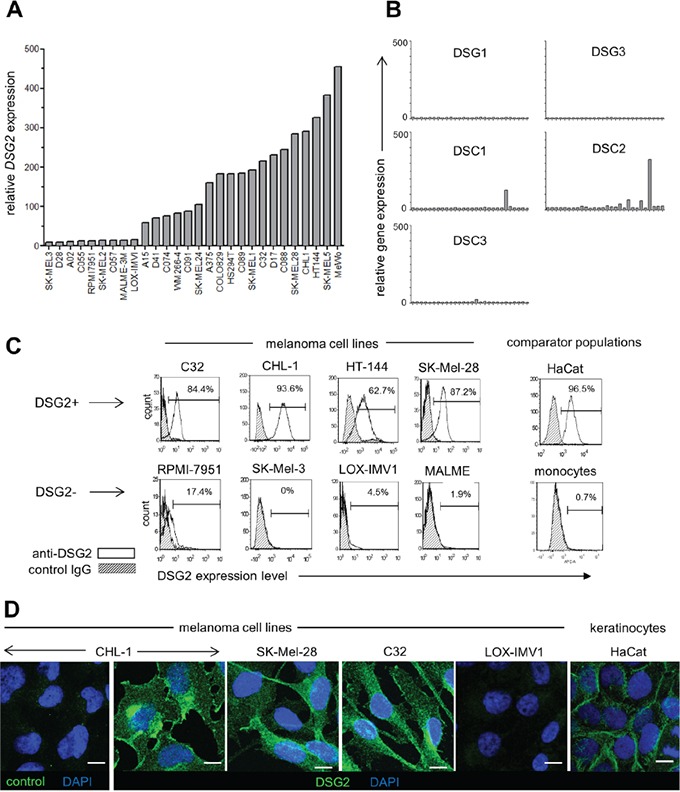
DSG2 is heterogeneously expressed by human melanoma cell lines **A.** Relative *DSG2* gene expression was determined by microarray analysis of 28 melanoma cell lines. **B.** For comparison, expression of other desmosomal cadherin genes was determined for the same panel of cell lines. **C.** Four each of *DSG2*-high and *DSG2*-low cell lines were selected for validation of microarray data by flow cytometry. Known positive (HaCat) and negative (monocyte) populations are shown for comparison. Values within histograms represent % positive for DSG2 within marker region after subtracting background. **D.** Immunofluorescence staining of permeabilized melanoma cells and HaCat keratinocytes with DSG2 or isotype matched control mAb, as indicated. Scale bar = 10μm

In a previous study [20], DSG2 expression was detected in 2/8 melanoma cell lines. To better understand the frequency of DSG2 expression on cultured melanoma cells, we performed gene expression microarray analysis on a panel of 28 human melanoma cell lines. As shown in Figure 1A, *DSG2* was clearly expressed in 68% of cell lines (19/28), and the levels of *DSG2* expression within positive lines was markedly heterogeneous. In contrast, expression of other desmosomal cadherins (*DSG1, DSG3, DSC1, DSC2, DSC3)* was negligible (Figure 1B), revealing that *DSG2* is unique within this gene family for its expression in a large proportion of melanoma cell lines. *In silico* analysis of data from The Cancer Cell Line Encyclopedia (CCLE) [25] confirmed these findings (Supplementary Figure S1). Thus, amongst a panel of 41 additional human melanoma cell lines, *DSG2* was broadly and heterogeneously expressed while the other desmosomal cadherins showed negligible expression.

To evaluate DSG2 protein expression in cell lines demonstrating high and low *DSG2* gene expression, four cell lines from each category were selected for validation (Figure 1C). Flow cytometry confirmed ubiquitous expression of DSG2 surface protein on each *DSG2*-high cell line, with a similar intensity of expression to that observed for desmosome-forming HaCat cells. In contrast, DSG2 protein was undetectable or very low in cell lines with low gene expression, similar to blood monocytes (which lack desmosomes and DSG2 expression; Ebert *et al*, submitted). In DSG2^+^ cell lines, immunofluorescence microscopy revealed diffuse distribution of DSG2 over cell membranes as well as intracellularly, particularly within perinuclear puncta (Figure 1D). This was in contrast to the localization of DSG2 at cell-cell junctions in HaCat keratinocytes, where DSG2 is an integral component of desmosomes, and the absence of DSG2 immunostaining in the LOX-IMV1 cell line, which lacks DSG2 gene and protein expression (as shown in Figure 1A and 1C). Together, these results demonstrate that (i) in contrast to other desmosomal cadherins, expression of DSG2 is relatively frequent amongst melanoma cell lines, (ii) differences in *DSG2* gene expression are also reflected at the level of protein expression and (iii) DSG2 protein displays a non-desmosomal distribution in melanoma cells.

### DSG2 is expressed in primary and metastatic melanoma tissue, but not in normal melanocytes

**Figure 2:**
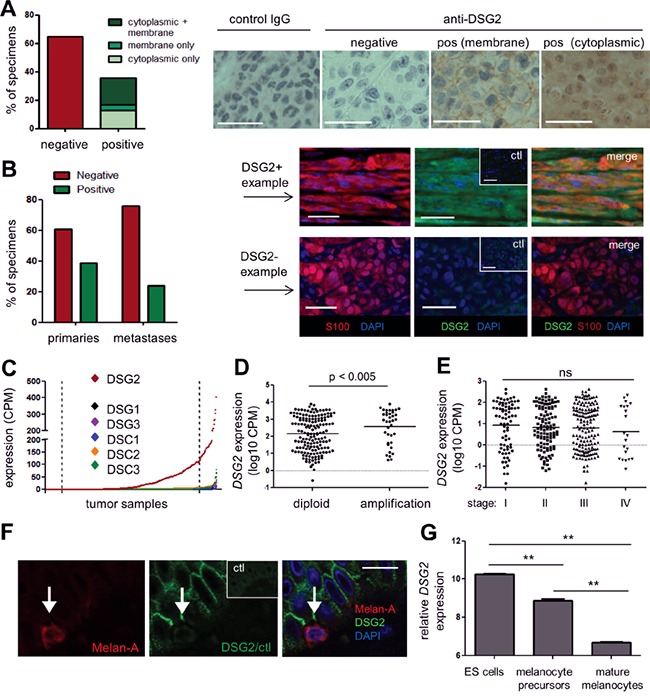
DSG2 is expressed in human primary and metastatic melanoma tissue **A.** DSG2 expression was examined in a metastatic melanoma TMA by immunohistochemistry (brown) with hematoxylin nuclear staining (blue). Bar graph summarizes results from 96 tumor samples; representative examples are shown on the right. **B.** DSG2 expression was examined in full-sized tumor tissue sections using immunofluorescence, including co-staining with S100 to confirm the identity of melanoma deposits. A summary of staining in primary vs metastatic tumors is shown on the left, and examples of a positive and a negative sample are shown on the right. Ctl; control IgG. **C-E.** RNA sequencing data obtained from TCGA was used to determine expression of desmosomal cadherin genes in melanoma samples (n = 427). (C) shows expression of each gene in a different color in scatterplot format, whereby each dot represents an individual tumor sample. Samples are arranged in order of intensity of expression, to aid visualization of the data. Dotted lines indicate upper and lower 10% cut-points used for subsequent analyses. (D) shows *DSG2* expression for samples with and without copy number amplification at the *DSG2* gene locus and (E) shows *DSG2* expression values for each disease stage (horizontal lines indicate mean). Groups were compared using Mann-Whitney (D) or Kruskal-Wallis (E) tests. **F.** Normal human skin was examined for DSG2 expression by immunofluorescence, using Melan-A to detect melanocytes; representative of n = 4 donors. **G.** *DSG2* expression in microarray dataset GSE54226, comparing ES cells and their derivatives induced to differentiate to melanocyte precursors and mature pigmented melanocytes (n = 2-3 clones of each; shown as mean ± SEM; ** p < 0.01 by ANOVA). Scale bars = 50μm.

There are conflicting reports regarding DSG2 expression in patient melanomas [20, 22, 23]. To resolve this, we undertook a comprehensive analysis of DSG2 expression in a large number of patient melanomas using two different anti-DSG2 mAbs. Initially, the 6D8 clone [26] was used to stain two tissue microarrays (TMAs) containing duplicate cores from 96 metastatic (Stage III/IV) melanomas with detection by immunohistochemistry. As shown in Figure 2A, 35% of tumors had clear DSG2 staining in both replicate cores compared to an isotype-matched negative control. Interestingly, the staining patterns observed varied markedly in both intensity and sub-cellular localization of DSG2, with 12% of positive samples demonstrating membranous staining, 35% cytoplasmic staining, and the remainder showing mixed membranous and cytoplasmic DSG2 expression.

To validate these results and compare DSG2 expression in primary versus metastatic disease, we next evaluated whole tissue sections using a different anti-DSG2 mAb, clone 10G11 [27]. A multi-color immunofluorescence approach was utilized for these experiments, to enable the identification of melanoma deposits via co-staining for the melanoma marker S100; this was particularly important when analyzing small primary tumors. Analysis of primary (stage I/II; n = 46) and metastatic (stage III/IV n = 25) tumors revealed DSG2 expression by S100^+^ melanoma cells in 39% of primary tumors and 24% of metastatic tumors, which was not a statistically significant difference (p = 0.294, Fisher's Exact test) (Figure 2B). Of note, the frequency of DSG2^+^ tumors observed in this analysis using the 10G11 mAb was very similar to the results obtained in Figure 2A using the 6D8 mAb, providing independent validation of the proportion of human melanomas that express DSG2.

We next investigated whether DSG2 expression varies temporally or spatially in patients with metastatic disease. Matched primary and metastatic tumors were obtained from eight patients, and multiple metastatic tumors were collected simultaneously from three further patients via rapid autopsy (Table 1). For the majority of patients (6/8), the expression of DSG2 did not vary between their primary and metastatic tumors. However, one patient had a DSG2^−^ primary tumor and a DSG2^+^ metastasis and a second patient had a DSG2^+^ primary and a DSG2^−^ metastasis. In the autopsy collections, one patient had uniformly DSG2^+^ disease while for the other two patients, only 5/6 and 2/5 of their metastatic tumors were DSG2^+^. Therefore, DSG2 can be heterogeneously expressed within individuals with metastatic melanoma. There was no association between site of metastasis and DSG2 expression in this small cohort.

**Table 1:** DSG2 expression in patient tissues can be spatially and temporally regulated

Patient	Tumors collected sequentially						
	Primary			Metastasis			
				DSG2		site	
1	−			−		lymph node	
2	−			−		lymph node	
3	−			+		dermis	
4	+			+		lung	
5	+			−		lymph node	
6	+			+		lymph node	
7	+			+		soft tissue	
8	+			+		lymph node	

Patient	Metastatic tumors collected at autopsy							
		#1	#2	#3	#4	#5	#6
CA006	DSG2	−	−	−	−	−	NA
	tumor	axilla	small	small	small	liver	
	site		bowel	bowel	bowel		
CA019	DSG2	+	+	+	+	+	−
	tumor	small	small	liver	liver	lung	lung
	site	bowel	bowel	(L lobe)	(R lobe)	(L)	(R)
CA023	DSG2	−	+	+	−	−	NA
	tumor	liver	liver	liver	lung	brain	
	site	(R lobe)	(central)	(L lobe)	(L)		

Tumors were obtained as either matched primary (Stage II) and metastatic (Stage III or IV) pairs (top section), or as multiple metastatic tumors collected at autopsy (lower section) and examined for DSG2 expression by immunohistochemistry (top) or immunofluorescence (lower). NA; tissue not available. +/−; DSG2 present or absent, respectively.

To support our analysis of DSG2 protein, we also analyzed RNA sequencing data from The Cancer Genome Atlas (TCGA). Of note, several recent publications have highlighted the significance of heterogeneity within tumor samples subjected to gene expression profiling. In particular, it is becoming increasingly recognized that the varying stromal content of tumors can dramatically influence the outcome of these analyses, including data within TCGA [28–30]. Because *DSG2* is normally expressed by keratinocytes, it was important to filter the input dataset to exclude any samples suspected to contain significant amounts of normal skin. Initially, we plotted the expression of three genes which are tightly restricted to the keratinocyte lineage (*IVL, KRT14 and BNC*) for the 473 melanoma samples within the TCGA RNA sequencing dataset (Supplementary Figure S2A). Each of these genes displayed an exponential distribution, being undetectable in the majority of samples but showing extremely high levels of expression in a small subset of samples. Moreover, as expected, the expression of these three keratinocyte genes was tightly correlated (Supplementary Figure S2B). On the basis of these results, we filtered the samples to exclude any with expression of all three genes >5CPM, or any two >20CPM. This filtering resulted in removal of 46 samples likely to have a high degree of contamination with normal skin, leaving 427 samples for downstream analysis.

The expression of *DSG2* and other desmosomal cadherin genes was determined within this filtered dataset. As shown in Figure 2C, *DSG2* was broadly and heterogeneously expressed, while other desmosomal cadherin genes (*DSG1, DSG3, DSC1, DSC2, DSC3*) were rarely expressed, and the mean expression level across all samples was significantly lower than for *DSG2* (p < 0.001 for all; Kruskal-Wallis test). Interestingly, analysis of copy number variation (CNV) predicted using the GISTIC algorithm at cBioPortal [31] for a subset of these samples revealed that 48/356 tumors (13.5%) displayed copy number amplification at the *DSG2* gene locus. Moreover, the level of *DSG2* mRNA was significantly increased in this subset of patients compared to samples without amplification (Figure 2D), suggesting CNV as one possible mechanism contributing to DSG2 up-regulation in melanoma, although non-genetic mechanisms are also clearly involved. In keeping with our immunostaining results, there were no differences in expression of *DSG2* between stages of disease progression in TCGA samples (Figure 2E).

To assess whether DSG2 is expressed by melanocytes (the cell of origin in melanoma), sections of normal adult skin were co-stained for DSG2 and the melanocyte marker melan-A. Of 25 individual melanocytes analyzed across 4 normal skin samples, none showed evidence of DSG2 expression (Figure 2F), in keeping with previous work [22]. To better understand DSG2 expression within the melanocyte lineage, we also performed *in silico* analysis of a published microarray dataset examining changes in gene expression during differentiation of human embryonic stem (ES) cells to mature melanocytes *in vitro* [32]. As expected from previous studies [33], pluripotent ES cells expressed high levels of *DSG2* (Figure 2G). However, this was reduced upon directed differentiation of ES cells to melanocyte precursors and further reduced upon differentiation to mature melanocytes.

Together, these data reveal that DSG2 (distinct from other desmosomal cadherins) is induced *de novo* in a subset of human melanomas and can be expressed throughout the disease course, but may be subject to temporal and spatial regulation within individual patients. Moreover, the expression of DSG2 in melanoma might represent re-activation of an embryonic pathway that is suppressed in normal adult melanocytes.

### Overexpression of DSG2 at the mRNA and protein level is associated with poor clinical outcome

**Figure 3:**
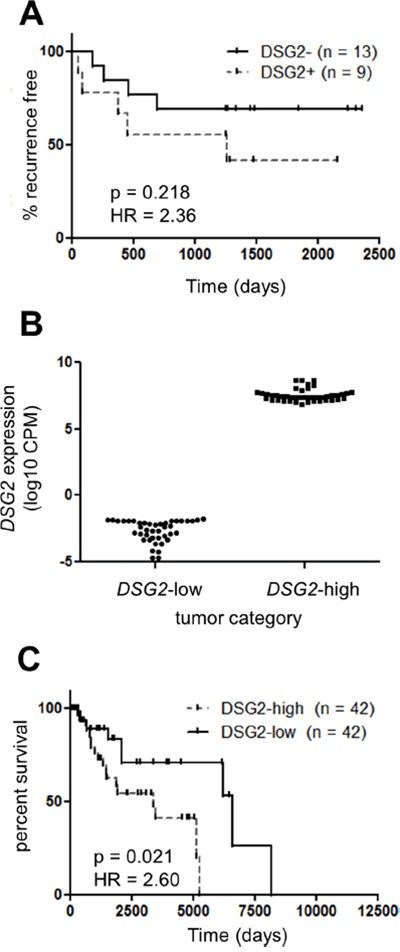
DSG2 over-expression in melanoma is associated with poor clinical outcome **A.** The rate of progression to metastatic disease was determined for 22 patients with Stage II (primary) melanomas. Patients were stratified into DSG2^+^ and DSG2^−^ subsets on the basis of immunofluorescence staining (see Figure 2B) and Kaplan-Meier analysis used to determine the time to tumor recurrence. **B-C.** TCGA *DSG2* gene expression data was used to identify patients within the top and bottom 10% of the *DSG2* expression range, as indicated by the dotted lines in Figure 2C. The level of *DSG2* gene expression for each group is shown in (B), while a Kaplan-Meier analysis of overall survival time from initial diagnosis is shown in (C).

To begin investigating the functional significance of DSG2 expression in melanoma, we correlated stage II patient survival with DSG2 protein expression (as determined in Figure 2B). Figure 3A shows that patients with DSG2^+^ stage II melanomas were more likely to develop metastatic disease compared to those with DSG2^−^ tumors (HR = 2.34), but this trend was not statistically significant in this small cohort (p = 0.22 log-rank test; n = 22). We therefore performed further analyses using gene expression and survival data from the much larger TCGA cohort. We stratified samples into *DSG2*-high and *DSG2*-low groups by setting cut points at the top and bottom 10% of the *DSG2* gene expression range (Figures 2C and 3B), and compared survival outcomes in these two groups. As shown in Figure 3C, the median overall survival of *DSG2*-high patients was 3379 days, compared with 6598 days in *DSG2*-low patients. This equated to a 2.6-fold increased rate of death (hazard ratio) for *DSG2*-high patients (p = 0.02, log-rank test). These results support the findings from our own cohort (Figure 3A) and posit DSG2 as a new prognostic marker in melanoma.

### DSG2 regulates tube formation by melanoma cells

**Figure 4:**
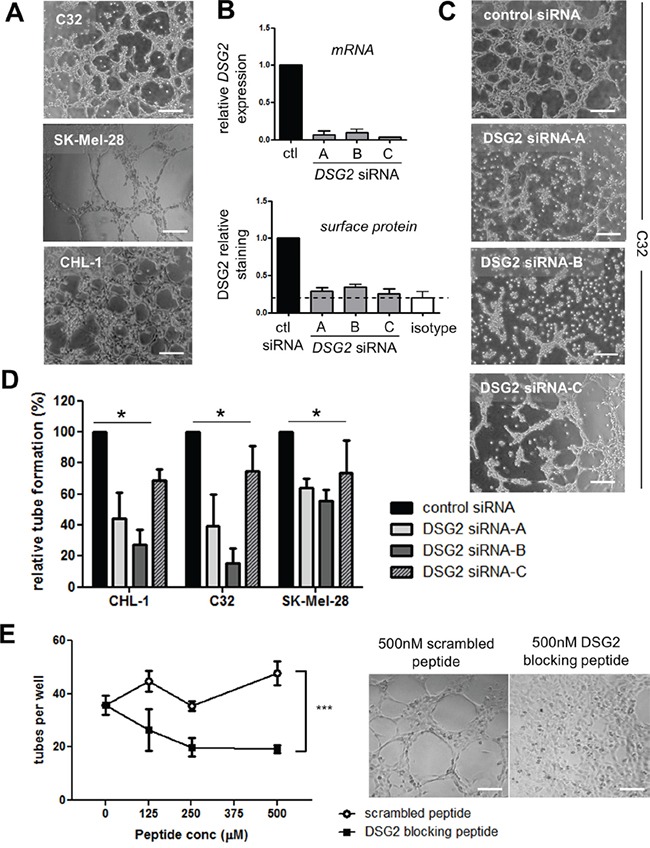
DSG2 regulates the formation of tube-like structures by melanoma cells on Matrigel **A.** Photomicrographs illustrate tube formation by three DSG2^+^ cell lines (C32, SK-Mel-28 and CHL-1) 4-6 hr after seeding on Matrigel. **B.** Validation of gene knockdown following treatment of the C32 cell line with *DSG2*-targeting or control siRNAs. The level of *DSG2* mRNA was quantified after 48 hr by qPCR (top), or DSG2 surface protein was quantified after 72 hr by flow cytometry (bottom). Graphs show mean ± SEM (normalized to control siRNA), pooled from 2 – 4 experiments. **C.** An example of the effect of *DSG2* knockdown on tube formation by C32 melanoma cells after 6 hr. **D.** The number of tubes formed per well was quantified for three melanoma cell lines. Data show mean ± SEM (normalized to control siRNA) pooled from 3-4 experiments; * p < 0.05 by ANOVA. **E.** DSG2 blocking peptide was added to CHL-1 cells prior to seeding on Matrigel, and tube formation after 3.5 hr was quantified as above. Graph on the left shows tube counts per well (mean +/− SEM, pooled from 4 experiments; *** = p < 0.001 by ANOVA). Images on the right show representative results at 500μM peptide. Scale bars = 200μm.

The results presented thus far demonstrate that DSG2 is overexpressed in a large fraction of melanomas compared to normal melanocytes, and that this overexpression is associated with poor clinical outcome. We therefore hypothesized that DSG2 regulates at least one aspect of melanoma cell biology that contributes to tumor growth or spread. Based on our previous studies revealing a role for DSG2 in vascular biology (Ebert *et al*, submitted), we reasoned that this key function for DSG2 in melanoma may be the promotion of VM. To address this possibility, three melanoma cell lines expressing high levels of DSG2 (C32, SK-Mel-28 and CHL-1; see Figure 1) were selected for detailed functional analyses. As shown in Figure 4A, all three cell lines formed an extensive network of tube-like structures within 6 hr of seeding on Matrigel, thus mimicking the behavior of ECs on Matrigel and demonstrating a high VM capacity of these cells [3, 4, 13]. To determine a role for DSG2 in regulating this process, gene knockdown was performed 48-72 hr prior to seeding cells on Matrigel. Each of three different *DSG2*-targeting siRNAs led to efficient reduction of both gene and surface DSG2 protein levels (Figure 4B). When these cells were seeded onto Matrigel, marked inhibition of tube formation was observed, with *DSG2*-knockdown cells forming fragmented networks or remaining in irregularly shaped clusters of cells (see examples in Figure 4C). Inhibition of tube formation upon *DSG2* knockdown was observed in all three cell lines (Figure 4D).

To further test the ability of DSG2 to regulate tube formation, we used a 10-amino acid competitor peptide to block DSG2 homotypic interactions. This peptide targets the DSG2 cell adhesion recognition (CAR) domain and has been previously demonstrated to block DSG2-mediated alveolar morphogenesis by epithelial cells [34] and colon carcinoma cell aggregation [35]. When this peptide was mixed with CHL-1 melanoma cells prior to seeding on Matrigel, striking inhibition of tube formation was observed compared to a scrambled control peptide (Figure 4E).

### Investigating the mechanism by which DSG2 regulates tube formation

Previous studies have shown that DSG2 can regulate several biological processes in addition to its canonical function in desmosomal adhesion, including proliferation and cell death in epithelial cells [36–38], actin cytoskeletal architecture in ECs [24] and melanoma cell migration [21]. We thus assessed the effect of DSG2 inhibition on these parameters in melanoma cells, to determine whether they could contribute to the observed reduction in tube formation.

**Figure 5:**
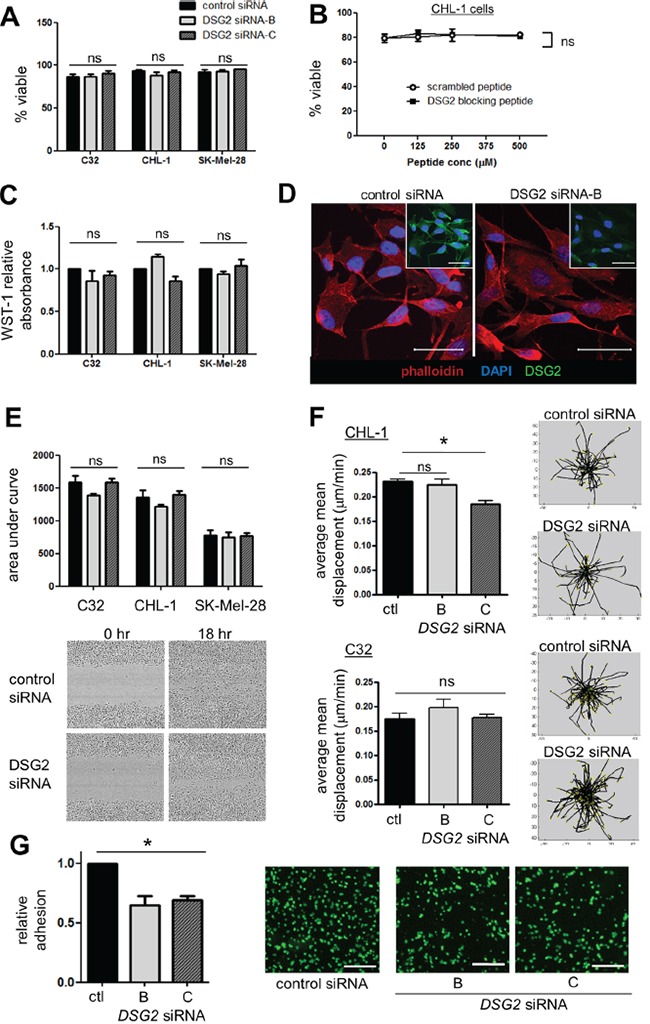
Effect of DSG2 inhibition on cell survival, proliferation, cytoskeletal structure, migration and adhesion **A-B.** Cell viability was assessed by 7-AAD staining following 48-72 hr siRNA-mediated knockdown (A) or 4 hr incubation with peptide (B). **C.** Cells were treated with siRNA for 48-72 hr, the cell proliferation reagent WST-1 added, and absorbance measured 90 minutes later. **D.** C32 cells transfected with control or *DSG2* siRNA were grown on coverslips for 48 hr, stained with DAPI (blue), rhodamine-phalloidin (red) and anti-DSG2 (green; insets) and analyzed by confocal microscopy. **E.** Scratch wound migration assays: wound size was measured every hour and time-course curves compared by determining area under the curve. Images show 0 hr and 18 hr time points using SK-Mel-28 cells. **F.** CHL-1 cells (top) or C32 cells (bottom) were seeded on Matrigel and cell migration during the first 2 hr of tube formation monitored by time lapse confocal imaging and quantified using the TLA program. Representative plots illustrate centrally-equalized migration tracks for individual cells within one field of view. **G.** Adhesion assays: Fluorescently-labeled C32 cells (treated with control or *DSG2*-targeting siRNA) were incubated for 15 min on top of a confluent monolayer of C32 cells, also treated with the same siRNA. After extensive washing in a parallel plate flow chamber, remaining adherent cells were quantified in 4 fields of view. Scale bars = 250μm. For all: bar graphs show mean ± SEM, n ≥3 experiments; ns - not significant; * p < 0.05 by ANOVA.

As shown in Figure 5A-5B, there was no change in cell viability after either *DSG2* knockdown (for 48-72 hr) or 4 hr incubation with DSG2 blocking peptide. Similarly, melanoma cell proliferation was unaffected by *DSG2* knockdown (Figure 5C). To determine whether DSG2 regulates cell morphology and cytoskeletal architecture, we performed phalloidin staining to detect polymerized F-actin structures (Figure 5D and Supplementary Figure S3). Analysis of stained cells by confocal microscopy confirmed DSG2 knockdown (insets) but revealed no consistent changes to cell shape, and the structure of the actin cytoskeleton was also largely unchanged. To measure cell migration, scratch wound assays were undertaken by creating a uniform scratch in confluent cell monolayers and then measuring wound thickness every hour, as cells migrate in to close the wound. When time-course curves were compared (by determining the area under the curve), we observed no detectable effect of DSG2 knockdown on migration of C32, CHL-1 or SK-Mel-28 cells (Figure 5E). To confirm these results in a different assay and investigate the behavior of individual cells during tube formation, we seeded CHL-1 cells on Matrigel and performed time lapse imaging and Time Lapse Analyzer tracking [39] to measure the distances travelled by cells during the first 2 hr of tube formation (Figure 5F and Supplementary Movies S1-S4). Cell migration on Matrigel was not affected by knockdown with DSG2 siRNA-B, although DSG2 siRNA-C (which routinely gave slightly more efficient knockdown; see Figure 4B), marginally reduced migration. We therefore repeated these experiments with C32 cells but found no effect of either siRNA. Thus, using two different assays, we could not reproduce the reported [21] increase in melanoma cell migration upon DSG2 knockdown. Note that, for each of these functional assays using *DSG2* knockdown, efficiency of knockdown was confirmed by flow cytometric analysis of DSG2 expression on the day of the assay (not shown).

Despite the lack of other desmosomal cadherins, DSG2 may nevertheless mediate melanoma cell adhesion in a desmosome-independent context, a possibility which has been previously proposed but not rigorously tested [20, 40]. We therefore measured the ability of C32 cells to adhere to each other, by generating confluent monolayers of cells (treated with control or *DSG2*-targeting siRNA) and incubating them for 15 mins with fluorescently-labeled cells also treated with the same siRNA, followed by stringent washing. Strikingly, despite the plethora of adhesion molecules expressed on melanoma cells, reducing expression of just DSG2 produced a significant reduction in cell-cell adhesion (Figure 5G, p < 0.01). Notably, time lapse imaging of tube formation on Matrigel also provided evidence of a role for DSG2 in regulating cell adhesion (Supplementary Movies S1-S4). Thus, cells with *DSG2* knockdown generally failed to form close attachments with adjacent cells, instead either spreading circumferentially on the Matrigel to form irregular patches (CHL-1) or remaining in small, loose clusters (C32). Together, these observations suggest that the major function of DSG2 in tube formation is to regulate cell-cell adhesion, thereby stabilizing and strengthening VM networks.

### DSG2 expression is associated with increased occurrence of VM and overexpression of VM-associated genes in human melanoma

**Figure 6:**
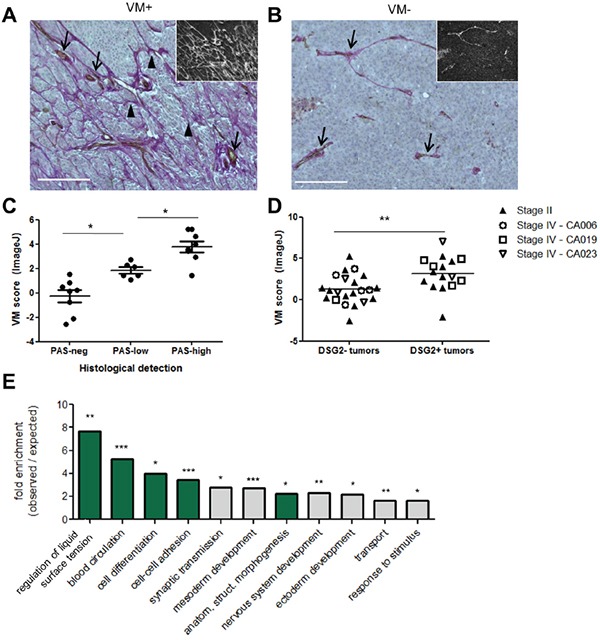
Expression of DSG2 in patient tumors is associated with increased VM Tissue sections were assessed for VM by co-staining with PAS (magenta) and anti-CD31 (brown) with hematoxylin counter-staining (blue). **A-B.** Examples of VM^+^ (A) and VM^−^ (B) tissues are shown. Main images depict colorimetric staining; insets show fluorescent images of the same field of view, for easier visualization of PAS^+^ networks. Scale bar = 200μM. **C-D.** VM was quantified using ImageJ (see Supplementary Materials and Methods). For (C), these scores were plotted separately for Stage II tumors falling into the categories of ‘PAS-neg’ (no PAS networks), ‘PAS-low’ (PAS networks covering <10% of area, or disconnected arcs only) and ‘PAS-high’ (extensive PAS networks) according to visual examination. Each symbol represents an individual tumor; * p < 0.05; *** p < 0.001 by ANOVA. For (D), VM scores were plotted separately for tumors identified as DSG2^−^ or DSG2^+^ by immunofluorescence (see Figure 2B). Each symbol represents an individual tumor, with Stage II tumors identified by solid symbols and the multiple tumors collected from Stage IV patients at autopsy identified by patient-specific open symbols; ** p < 0.01 by unpaired t-test. **E.** TCGA RNA sequencing data was used to identify tumors within the top and bottom 10% of the *DSG2* expression range, as shown in Figure 3B. A differential gene expression analysis was performed and the list of genes overexpressed by the *DSG2*-high group (see Supplementary Table S1) was subject to Gene Ontology analysis. Graph shows significantly enriched GO-Slim terms; where multiple terms within a hierarchy were significantly enriched, only the most specific subclass is shown. Terms with relevance to VM are highlighted in green; *** p < 0.001, ** p < 0.01, * p < 0.05.

Our mechanistic studies using cell lines revealed an important role for DSG2 in promoting melanoma cell tube formation on Matrigel, an *in vitro* correlate of VM. We therefore next assessed a potential link between DSG2 expression and VM occurrence in patient melanomas. To quantify VM, tissue sections were stained with anti-CD31 using immunohistochemistry (to detect ECs) in combination with Periodic Acid-Schiff (PAS). This latter reagent stains basement membrane structures (including those lining both VM channels and normal blood vessels) magenta, and is routinely employed to detect VM networks in tumors [10–14]. Examples of tumors with pronounced VM (i.e. branching networks of PAS^+^ structures which lack CD31 staining) and with no detectable VM (lack of PAS^+^ networks) are shown in Figures 6A and 6B, respectively.

To quantify the density of VM networks, we took advantage of the fact that PAS staining also produces a red fluorescent signal [41, 42] (Figure 6A-6B insets), in addition to the more frequently reported colorimetric reaction. Fluorescent images of each tissue were first analyzed in ImageJ using the ‘Tubeness’ algorithm to detect branching networks [43]. Networks co-staining for CD31 were then subtracted from the image to enable calculation of the total area occupied by PAS^+^CD31^−^ networks, as described in Supplementary Materials and Methods. This approach for quantifying VM, which is usually described qualitatively [10–14], was first validated by assessing each tumor histologically according to the criteria of Warso *et al* [11] and comparing these results to the calculated VM score (Figure 6C). As expected, tumors with extensive PAS^+^ CD31^−^ networks had a significantly higher VM score than those which lacked these structures or had very limited PAS patterning. Notably, when this quantitative approach was used to compare VM levels in DSG2^+^ and DSG2^−^ tumors (defined according the results of Figure 2 and Table 1), significantly higher VM scores were observed in DSG2^+^ melanomas compared to DSG2^−^ melanomas (mean 3.24 *vs* 1.42; p < 0.01; Figure 6D). Thus, DSG2 overexpression is associated with increased VM in human melanoma.

Finally, we performed differential gene expression analysis of TCGA data to identify genes overexpressed in *DSG2*-high compared to *DSG2*-low melanomas. A total of 286 genes was significantly overexpressed (>2-fold; false discovery rate (FDR) < 0.005) in the *DSG2*-high group (Supplementary Figure S4 and Supplementary Table S1), while 139 genes were underexpressed. Overexpressed genes included those associated with EC function (e.g. *NRP1, EDIL3, EDNRA, AGTR1*), collagen production (including basement membrane components *COL4A3* and *COL4A5*) and a progenitor status (e.g. PROM1), as well as several proteases involved in modifying ECM (e.g. *MMP9, MMP1, PRSS21, TMPRSS2* and *PLAU*). *DSG2*-high tumors also demonstrated overexpression of three members of the *SERPIN* gene family (*SERPINB2, SERPIND1* and *SERPINE1*). These genes all encode serine protease inhibitors which regulate blood clotting, an activity thought to be essential for the generation of perfused VM structures. Of note, SerpinE2, another Serpin family member which regulates clotting, has recently been shown to be critical for VM activity of breast cancer cells [18]. When the list of overexpressed genes was subject to gene ontology analysis using PANTHER [44], the annotations included ‘blood circulation’, ‘anatomical structure morphogenesis’, and ‘cell-cell adhesion’, all terms relating to the generation and function of vascular structures (Figure 6E). Interestingly, several annotations associated with embryogenesis were also enriched, in keeping with the concept that VM is induced in tumors that revert to an embryonic-like state [3, 4]. Thus, melanoma cells expressing high levels of *DSG2* also express elevated levels of VM-associated genes, suggesting that DSG2 is part of a broader genetic program which regulates VM in tumors.

## DISCUSSION

VM is increasingly accepted as an important promoter of cancer growth and metastasis. Here, we have identified DSG2 as a novel regulator of VM in human melanoma. Compared to normal melanocytes, DSG2 was overexpressed in a substantial fraction of human melanoma tissues and cell lines, and its expression in patient tumors was associated with enhanced VM, an enrichment of VM-associated genes and reduced overall survival. *In vitro* studies confirmed a direct role for DSG2 in the formation of VM-like structures. On the basis of these findings, we propose that DSG2 is induced during tumorigenesis in a large subset of melanoma patients and functions to promote the formation of VM networks, which in turn may help drive tumor growth and progression.

Historically, the principal function ascribed to DSG2 has been the formation of desmosomal adhesion structures in epithelia and myocardium. However, DSG2 is increasingly recognized to also play important biological functions unrelated to desmosome formation [20, 21, 24, 36–38]. Most notably for the present study, recent data implicate DSG2 in regulating the angiogenic activity of ECs, whereby loss of function disrupts tube formation on Matrigel and *in vivo* neoangiogenesis, and is associated with defective angiogenesis in patients with systemic sclerosis [24] (Ebert *et al*, submitted). Here, we demonstrate that DSG2 fulfils a related function in melanoma, by promoting VM. This VM-promoting activity appears to represent yet another function for DSG2 which is independent of desmosomes, as its distribution in melanoma was not typical of desmosomal localization, and melanoma cells generally lacked co-expression of desmocollins, which are required to form functional desmosomes [45].

The formation of VM channels in tumors, or the *in vitro* correlate of this process – tube formation on Matrigel [3, 4, 13] – are highly complex processes. It was therefore important to determine the precise activity which DSG2 controls in melanoma cells during tube formation. Many of the non-desmosomal functions ascribed to DSG2 in various cell types could be expected to impact on the VM capacity of melanoma cells. However, in contrast to epithelial cells [36–38], we did not find any role for DSG2 in regulating the viability or proliferation rate of melanoma cells, at least following short-term loss of function. These results are in keeping with findings of a previous study in melanoma cells [21]. And while DSG2 has been suggested to regulate the actin cytoskeleton in ECs [24], *DSG2* knockdown did not have a similar effect in cultured melanoma cells. It remains possible, however, that DSG2 could regulate other cytoskeletal structures in these cells, or be required for active cytoskeletal rearrangements during tube formation. Finally, using two different approaches, we did not detect any change in melanoma cell migration following *DSG2* knockdown, which is in contrast to a previous study [21] for reasons that are presently unclear. On the other hand, *DSG2* knockdown did significantly reduce the ability of melanoma cells to adhere to each other, suggesting that DSG2 promotes VM by mediating intercellular adhesion, presumably in a non-desmosomal context. In support, live imaging of tube formation in the absence of DSG2 revealed that cells underwent initial migration and positioning similar to control cells, but at later time-points appeared unable to form the intimate cell contacts required for generation of a branching network.

VM is thought to occur when tumor cells revert to an undifferentiated, embryonic-like state, endowing them with the functional plasticity to mimic the behavior of ECs [3, 4]. In keeping with this concept, several proteins that play a key role in embryogenesis, such as Nodal and Notch4, can be re-expressed in aggressive melanoma and play a functional role in the VM process [46]. We found that *DSG2* is expressed at high levels by ES cells and down-regulated upon differentiation to mature melanocytes, but then re-expressed in a subset of melanomas. Furthermore, analysis of TCGA data revealed that melanomas overexpressing *DSG2* are also enriched in genes associated with embryogenesis. We therefore propose that DSG2 expression is induced in melanoma as part of a genetic program resulting in re-expression of embryonic genes, endowing cells with the functional plasticity required to undergo VM. Interestingly, although DSG2 clearly plays an important role in regulating VM, it is not absolutely required for this process, as we have identified several melanoma cell lines which lack DSG2 expression and yet can still form tubes on Matrigel (unpublished findings). In addition, not all tumors with evidence of VM expressed DSG2. It is likely that DSG2 is one of several cadherins which can contribute to VM, and indeed, previous studies have implicated VE-cadherin in this process [47, 48]. Furthermore, other members of the desmosomal cadherin family were expressed at low level in a small number of TCGA tumors, and *DSG1, DSC2* and *DSC3* were all significantly enriched in the *DSG2*-high tumor subset. It would be worthwhile determining in future studies whether these molecules also play a role in VM, and whether there is a degree of functional redundancy, or even cooperativity, amongst family members.

The importance of VM to melanoma pathogenesis is suggested by its close association with poor survival and increased metastasis in at least five separate human studies [10–14]. We found that DSG2 overexpression in human melanoma is associated with enhanced VM, increased expression of VM-associated genes and reduced progression-free and overall survival. In addition, DSG2 directly regulated VM activity (i.e. tube formation) *in vitro*. These data suggest that therapeutic targeting of DSG2 in melanoma might block the formation of VM networks. On the basis of previous studies, we hypothesize that this would in turn reduce tumor growth and metastasis. Given the established role of DSG2 in normal tissues [19], inhibiting DSG2 in patients could be expected to result in some level of toxicity. However, previous studies suggest that targeting DSG2 in patients is feasible. A small recombinant protein (JO-4) shown to disrupt DSG2-mediated adhesion was well tolerated in primate pre-clinical models [49], while clinical trials targeting the closely related N-cadherin have revealed an excellent safety profile for this approach with no dose-limiting toxicities, despite extensive expression of N-cadherin on normal tissues [50]. These studies raise the possibility that DSG2 could be therapeutically targeted in melanoma with acceptable toxicity, possibly using the JO-4 reagent, an antibody-drug conjugate, or a variant of the peptide inhibitor we used in Figure 4E. Importantly, these approaches would be expected to reduce both melanoma cell-mediated VM and EC-mediated angiogenesis [24] (and Ebert *et al*, submitted), and thereby provide more complete inhibition of tumor vascular networks than is achievable using current anti-angiogenesis therapeutics [2].

In summary, our observations reveal DSG2 as a novel regulator of VM in human melanoma and a marker of prognostic significance in this disease. Our data also add weight to evidence that DSG2 is unique amongst the desmosomal cadherins in its expression and function outside the context of desmosomes. Additional studies are warranted to further explore the importance of DSG2-induced VM to melanoma pathogenesis, and to evaluate this molecule as a new therapeutic target in melanoma.

## MATERIALS AND METHODS

### Cell culture

Melanoma cell lines were obtained from American Type Culture Collection or the Australasian Biospecimen Network-Oncology cell line bank at the Queensland Institute for Medical Research (QIMR) and their identity confirmed by PCR-based short tandem repeat (STR) analysis. Cells were cultured in RPMI containing 10% FCS (‘RF-10’; melanoma cells) or DMEM containing 10% FCS (HaCat cells) under standard conditions (37°C, 5% CO_2_), and periodically confirmed negative for mycoplasma using MycoAlert (Lonza). Cultures were routinely passaged using trypsin, although prior to functional assays, cells were instead detached with 10mM EDTA to prevent cleavage of surface molecules.

### Functional assays using melanoma cell lines

Tube formation assays were performed on Growth-Factor Reduced Matrigel from Falcon using Ibidi Angiogenesis culture slides. Tube-like structures (defined as elongated multi-cellular structures between groups of cells that are intimately joined, not just loosely aligned) were counted in a blinded manner. Scratch wound assays were performed using a WoundMaker™ and IncuCyte ZOOM^®^ (Essen BioScience) according to the manufacturer's instructions. Proliferation assays were conducted using Cell Proliferation Reagent WST-1 (Roche). To perform adhesion assays, Calcein-labeled and siRNA-treated cells were incubated for 15 min on a confluent monolayer of C32 cells (treated with the same siRNA), followed by washing and quantification of bound fluorescent cells. Full details of each assay are in Supplementary Materials and Methods.

### DSG2 gene knockdown using siRNA

‘Trilencer-27’ siRNA duplexes were purchased from Origene. Transfection was performed using Lipofectamine RNAiMax (Thermo Fisher) according to the manufacturer's protocol, using 1nM siRNA. On the basis of time course experiments, C32 and SK-Mel-28 cells were used in experiments 72 hr after knockdown, while CHL-1 cells were used 48 hr after knockdown. Knockdown efficiency was assessed by flow cytometry as a quality control check prior to performing functional assays.

### DSG2 function-blocking peptide

The DSG2 ectodomain competitive inhibitor peptide and scrambled control peptide were custom synthesized and purified by HPLC to ≥95% by Mimotopes (Clayton, VIC, Australia). Freeze-dried peptides were reconstituted in PBS and aliquots stored at −80°C. Sequences were: DSG2 peptide: LTGYALNARG; scrambled control peptide: YTRLGANLAG, based on Runswick *et al* [34]. Peptide was pre-mixed with melanoma cells prior to seeding on Matrigel.

### Gene expression microarray analysis of melanoma cell lines

Cells were harvested when 50–80% confluent. RNA was extracted (QIAGEN RNeasy kit) and *in vitro* transcribed and biotin labelled cRNA was fragmented and hybridized to Affymetrix 1.0ST expression array as per manufacturer's instructions. GeneChips were washed and stained in the Affymetrix GeneChip Fluidics Station 450 using standard Fluidics Protocol (Fluidics protocol FS450_007) and scanned using the Affymetrix 7G Scanner. The data was RMA normalized using the R Bioconductor affy package.

### RNA extraction, reverse transcription and quantitative PCR (qPCR)

RNA was extracted from cells, reverse transcribed and subject to real-time PCR according to standard protocols; details are in Supplementary Materials and Methods.

### Flow cytometry

Cells were stained with anti-DSG2 (clone 6D8; Thermo Fisher) or isotype-matched control antibody (clone MOPC-21; BD) at 5μg/ml for 20 min at 4°C, incubated with secondary antibody, washed and fixed in paraformaldehyde. All staining steps were performed in complete medium containing FCS, as this was found to produce optimal staining for DSG2 (Sun *et al*, manuscript in preparation). Samples were processed using a BD Accuri flow cytometer and data analyzed using FSC Express (De Novo software). Cell viability was determined by staining with 7-Amino-Actinomycin D (7-AAD Staining Solution; BD Biosciences) at a 1/20 dilution for 10 min prior to flow cytometric analysis.

### Patient tissues and TMAs

Formalin-fixed paraffin embedded (FFPE) melanoma tissues for immunohistochemistry analysis (including TMAs) were obtained from SA Pathology (Adelaide, Australia). Their use was approved by the Human Research Ethics Committee of the Royal Adelaide Hospital. TMAs were created using an Advanced Tissue Arrayer (Chemicon International). Human FFPE melanoma and normal tissues for immunofluorescence analysis were obtained from TissuPath and from established cohorts (the Melbourne Melanoma Project [51] and the CASCADE rapid autopsy program [52]), in accordance with the Peter MacCallum Cancer Centre Human Research Ethics Committee approved protocol 10/02.

### Immunofluorescence and immunohistochemistry

For staining of cell lines, cells were seeded onto coverslips and stained with anti-DSG2 (clone 6D8) together with rhodamine-phalloidin. For tissue analysis, sections were subject to heat-mediated antigen retrieval followed by either immunofluorescence staining using anti-DSG2 (clone 10G11; LSBio) and anti-S100 (clone Z0311; Dako); or immunohistochemistry using anti-DSG2 (clone 6D8) or anti-CD31 (rabbit polyclonal; Bethyl Laboratories) together with Periodic-Acid Schiff (PAS). Detailed protocols are in Supplemental Materials and Methods.

### Bioinformatics analysis of publically available datasets

Analysis of microarray data generated by Mica *et al* [32] was performed by analyzing Gene Expression Omnibus (GEO) dataset GSE45223 using the GEO2R analysis tool (http://www.ncbi.nlm.nih.gov/geo/geo2r/). Data are pooled from 2 ES cell clones and 3 clones each of melanocyte precursors and mature melanocytes. The Cancer Cell Line Encyclopedia dataset [25] was interrogated by analyzing GEO dataset GSE36133 using GEO2R. To analyze TCGA data, RNA sequencing (RNA-seqV2) and clinical (Biotab) data were downloaded from the Data Portal at http://cancergenome.nih.gov/. Data were analyzed in Bioconductor, using the edgeR package to perform differential gene expression analysis [53].

### Statistics

Statistical analysis was performed using GraphPad Prism v5.04. Survival analysis was conducted using Kaplan-Meier plots with log-rank tests. Other analyses were performed using unpaired Student's t-test (except where results were normalized to an internal control, in which case a paired t-test was used) or 1-way ANOVA as appropriate. Bonferroni post-tests were used to compare individual groups. For data with a non-normal distribution, Mann-Whitney and Kruskal-Wallis tests with Dunn's post-test were used instead. Results with p < 0.05 were considered significant, and are indicated on graphs according to the reporting summary in Prism v5.04, whereby * = p < 0.05 but > 0.01; ** = p < 0.01 but > 0.001 and *** = p < 0.001.

## SUPPLEMENTARY DATA












