# Supplementary material for: Desmoglein 2 promotes vasculogenic mimicry in melanoma and is associated with poor clinical outcome
Source: Oncotarget. 2016 Jun 22;7(29):46492–508. doi: 10.18632/oncotarget.10216 (PMC5216812; doi:10.18632/oncotarget.10216)
Supplement: Supplementary file 1 [file oncotarget-07-46492-s001.pdf]

Desmoglein 2 promotes vasculogenic mimicry in melanoma and is associated with poor clinical outcome

SUPPLEMENTARY DATA

Functional assays using melanoma cell lines

For tube formation assays, Growth Factor-Reduced (GFR) Matrigel (Falcon) was aliquoted into a 'µ-slide Angiogenesis' culture slide (Ibidi), allowed to gel and then melanoma cells were added and the slide incubated at 37°C with 5% CO_2 for 3-6 hrs. The optimal cell density and incubation time was determined empirically for each cell line and is indicated in the figure legends. Images were digitally captured on an IX70 microscope with F-view CCD camera (Olympus). The number of tube-like structures (rigorously defined as elongated structures between groups of cells that are intimately joined, not just loosely aligned) within each image were counted in a blinded manner. For live imaging, µ-slides were prepared as above and incubated at 37°C with 5% CO_2 for 7hr in a Yokogawa CV1000 confocal scanner box, collecting images every 5 min. Cell movement was quantified using Time Lapse Analyzer (39) to track migration during the first 2hr of the assay.

To perform scratch wound assays (for measurement of cell migration), melanoma cells were transfected with siRNAs as described in *Materials and Methods* and wounds created in confluent monolayers of cells in a 96 well plate using a WoundMaker™ (Essen Bioscience) according to the manufacturer's instructions. The plate was placed in an IncuCyte ZOOM® (Essen BioScience) and images were taken every hour for ≥24 hours, using the 10X objective. Wound area was assessed using the MRI Wound Healing Tool macro in ImageJ. A graph depicting the percentage of wound size relative to starting size over time was generated and results quantified as area under the curve using GraphPad Prism.

Proliferation assays were performed by transfecting cells with siRNAs for 48-72 hours and then adding Cell Proliferation Reagent WST-1 (Roche) for 90 min at 37°C. After 1 min on an orbital mixer, absorbance was measured using an Epoch™ microplate spectrophotometer (BioTek) at 450nm.

To perform adhesion assays, C32 cells (± *DSG2*-targeting or control siRNA) transfected 48 hours prior were cultured at $1x10^6$ cells/35mm x 10mm dish (Corning, Sigma-Aldrich) to form a confluent monolayer. Twenty-four hours later, $1x10^6$ of 72 h-transfected C32 cells (± *DSG2*-targeting or control siRNA) were labelled with 0.5 µM Calcein-AM (eBiosciences) and incubated on top of the confluent monolayer for 15 min after which time the non-adherent cells were washed with Hanks Balanced Salt Solution (Sigma-Aldrich) using a parallel plate flow chamber system (GlycoTech Corporation, Gaithersburg, MD, USA) with a flow rate of 0.156 mL/min for 5 min. Four random fields of view were taken across the gasket window using an inverted epifluorescence microscope Olympus IX71 and a 4x/0.10 objective. Adherent fluorescent cells were quantified using ImageJ.

RNA extraction, reverse transcription and quantitative PCR (qPCR)

RNA was extracted from cell pellets using the RNeasy Micro Plus kit (Qiagen) according to the manufacturer's protocol. Reverse transcription was performed on 1µg of purified RNA using a poly-T primer and Superscript III enzyme (Life Technologies). PCR primers were designed for human DSG2 (F-5'-CCATTGCGGAAAGGGCGCCA-3' and R-5'-GGCAGAAATGATGGCACCACCTTGT-3') using Primer Blast (NIH, MD, USA) and synthesized by GeneWorks (Hindmarsh, SA, Australia). Quantitative PCR amplification was performed using QuantiTectTM SYBR Green master mix (Qiagen) on a Rotor-Gene thermocycler (Corbett Research, NSW, Australia) with reaction parameters: 15 minutes at 95°C, then cycling of 10 seconds 95°C, 20 seconds 55°C and 30 seconds 72°C; for 45 cycles followed by a melt phase. Raw data were analyzed using Rotor-Gene Analysis Software version 6 (Corbett Research). Relative gene expression levels were calculated using standard curves generated by serial dilutions of cDNAs and normalized to the human house-keeping gene CycA (F-5'GGCAAATGCTGGACCCAA CACAAA-3', R-5'CTAGGCATGGGAGGGAACAAG GAA3').

Immunofluorescence and phalloidin staining of cell lines

Immediately after transfection with siRNA, cells were seeded onto coverslips pre-coated with fibronectin. After 48-72 hours, cells were fixed with 4% paraformaldehyde for 10 minutes, permeabilized with 0.1% Triton® X-100 (Merck Milipore) for 10 minutes and blocked with 5% human AB serum (Invitrogen) for 30 minutes (all at RT). Cells were then incubated with anti-DSG2 (5µg/ml; clone 6D8) or control mouse IgG1

(5µg/ml; clone MOPC-21) at 4°C overnight, washed and incubated with a mixture of Alexa Fluor 488 goat anti-mouse IgG (Molecular Probes) and rhodamine-phalloidin (Thermo Fisher) for 1hr at RT. After washing, coverslips were mounted onto glass slides using ProLong Gold® with DAPI (ThermoFisher). Imaging was performed on an LSM 710 confocal laser scanning microscope (Zeiss) using the 40x/1.0 DIC W Plan-Apochromat objective.

Immunofluorescence staining of FFPE tissue sections

Tissue sections were dewaxed and subject to heat induced antigen retrieval in a Pascal pressure cooker (Dako, Denmark) at 120°C for 3 minutes in EDTA (pH 9) antigen retrieval buffer (Leica). After cooling, sections were washed in 0.02% TBST 3 times for 7 minutes each and then blocked in 1% BSA/TBS for 30 minutes at RT before being incubated with primary antibodies diluted in 1% BSA/TBS: DSG2 (clone 10G11; LSBio) neat and S100 (clone Z0311; Dako) at a dilution of 1:2000, or with isotype controls (Invitrogen, mouse IgG1) overnight at 4°C. Sections were then washed in 0.02% TBST as above, followed by incubation with secondary antibodies (goat anti-mouse Alexa Fluor 488 IgG1 and goat anti-rabbit Alexa Fluor 633 IgG; Invitrogen) at a dilution of 1:400. Sections were washed in 0.02% TBST and counterstained with DAPI at a dilution of 1:500 in 1%BSA/TBS for 10 mins at RT. After washing in MQ water, sections were mounted in fluorescent mounting medium (Dako) prior to imaging on an Olympus BX-51 microscope mounted with a Spot RT3 digital camera (Diagnostic instruments).

Immunohistochemistry and PAS staining of tumor tissue

Slides were dewaxed and rehydrated prior to pressure cooker antigen retrieval for 10 min in pH 9.0 Tris/EDTA buffer (for DSG2 staining) or heat-mediated antigen retrieval in pH 6.0 citrate buffer (for CD31 staining). After cooling, sections were incubated for 60 min at RT with primary mAb against DSG2 (20µg/ml; clone 6D8; Thermo Fisher) or an isotype matched negative control at the same concentration (clone ICIGG1; Abcam); or with rabbit polyclonal anti-CD31 (2µg/ml, Bethyl Laboratories) at 4°C overnight. Primary antibody binding was revealed using the polymer system ADVANCE™ HRP (Dako) according to the manufacturer's recommendations. DSG2-stained sections were immediately counterstained using Mayer's hematoxylin and mounted in DPX, while CD31-stained sections were further stained using a PAS staining kit from Merck Millipore according to the manufacturer's instructions, before hematoxylin counterstaining and mounting. Colorimetric staining patterns were imaged using an EVOS XL system (Thermo Fisher), while fluorescence emission from PAS staining was photographed on an Olympus IX71 epifluorescence microscope equipped with XM10 camera using the 4X objective. Multiple fields of view were captured covering the majority of the tissue and images analyzed in ImageJ as described below.

Quantification of VM networks in tissue sections using ImageJ

Images captured as TIFF files were processed in ImageJ as follows, using polygon regions to define the tumor area where necessary (i.e. if the tumor didn't fill the entire field of view, or to exclude areas of necrosis or stroma).

1. Color images to detect CD31 staining

a) Each image was split into red, green and blue channels; the red and green channels were discarded

b) The threshold of the blue channel was adjusted to a value which accurately identified the areas of DAB staining. The same value (max 70) was used for 98% of images, although for the remaining 2%, a higher value of (max 100) had to be used, to account for poor color segmentation from the red channel

c) The 'measure' tool was used to determine the % of area above the threshold

d) For each tumor, the mean value of multiple images was calculated

2. Fluorescent images

a) Fluorescence emission was detected in the orange channel and captured as black and white images

b) Each image was pre-processed using the 'Tubeness' plugin using a sigma value of 0.01, and the resulting 32-bit image converted to an 8-bit image

c) The threshold was adjusted to a value which accurately identified the fluorescent PAS networks with the 'dark background' option selected. For the majority of samples (83.7%), this was (min 70, max 255), although for remainder, this was increased to (min 100, max 255) to account for higher tissue autofluorescence

d) The 'measure' tool was used to determine the % of area above the threshold

e) For each tumor, the mean value of multiple images was calculated

3. Final VM score: a single VM score was calculated for each tissue section using the following formula: (mean % area of PAS staining) – (mean % area of CD31 staining).

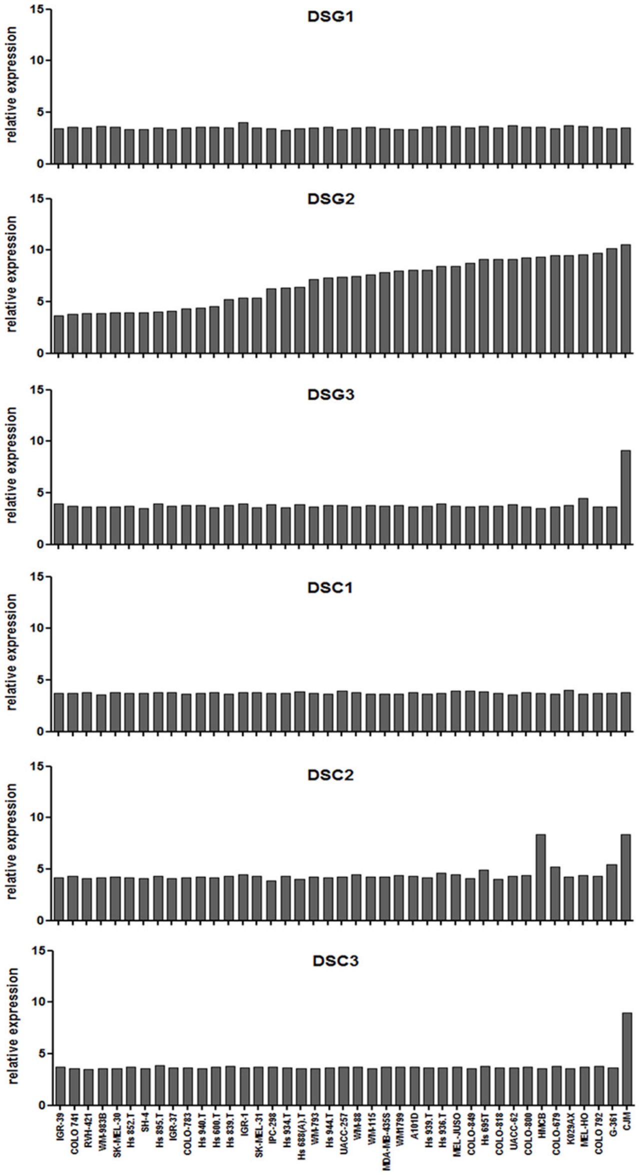

Supplementary Figure S1: DSG2 is broadly expressed by melanoma cell lines in comparison to other desmosomal cadherins. The Cancer Cell Line Encyclopedia (CCLE) gene expression microarray dataset (GSE36133) was interrogated using GEO2R to generate relative expression values for each of the melanoma cell lines in the database, after filtering to remove those already assessed in our own analysis (n = 41).

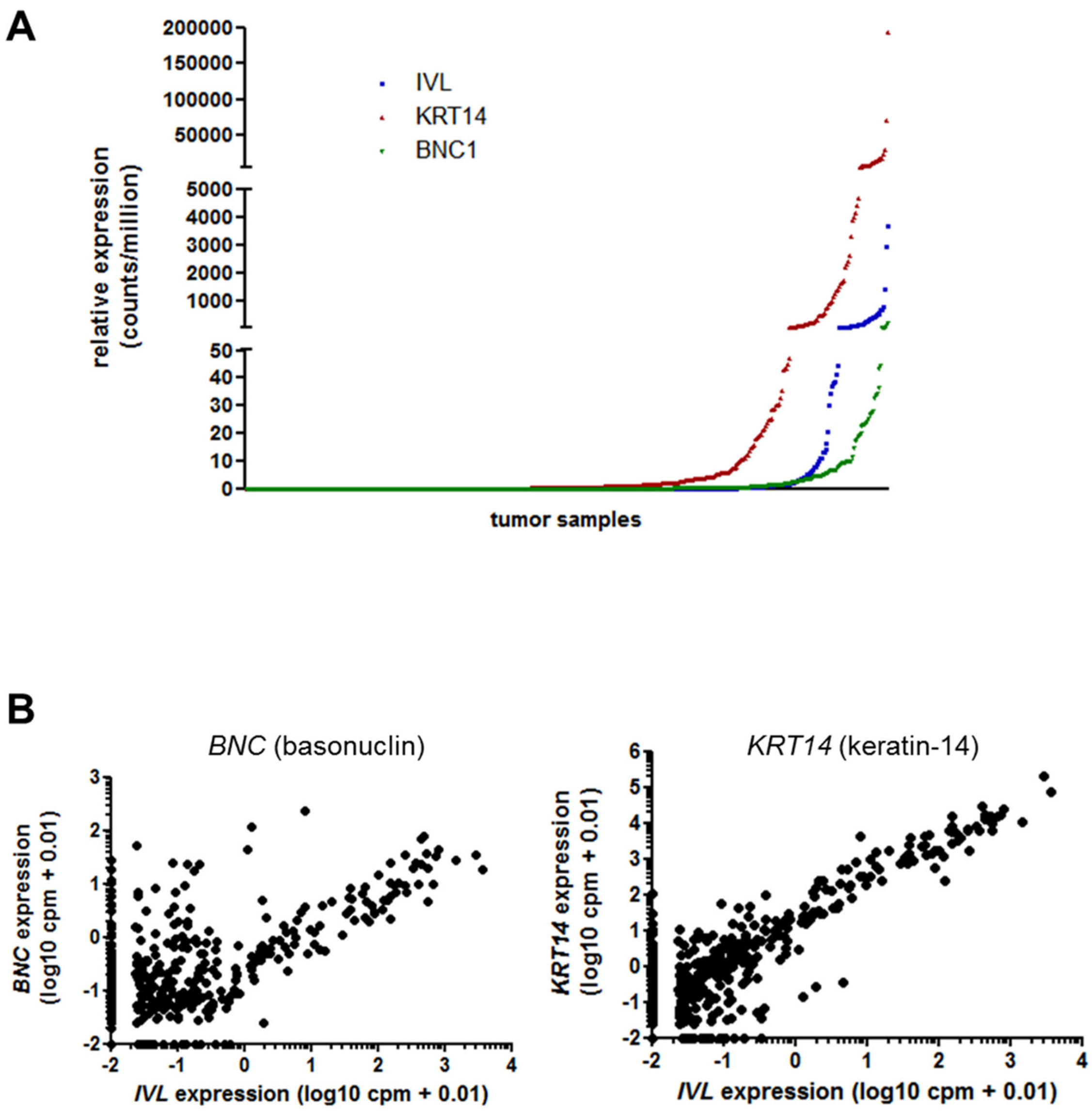

Spearman's correlation coefficient*

	IVL	KRT14	BNC1
IVL	-	0.7277465	0.4778882
KRT14	0.7277465	-	0.4611479
BNC1	0.4778882	0.4611479	-

* P-value < 0.0001 for all

Supplementary Figure S2: Expression of keratinocyte-restricted genes within TCGA melanoma samples. RNA sequencing data obtained from TCGA was used to determine expression of the keratinocyte lineage genes *IVL, KRT14* and *BNC* in melanoma samples (n = 473). **A.** shows expression of each gene in a different color in scatterplot format, whereby each dot represents an individual tumor sample. Samples are arranged in order of intensity of expression, to aid visualization of the data. **B.** shows the expression value for *IVL* for each sample plotted against the expression value for either *BNC* (left) or *KRT14* (right) from the same sample. Note that the value 0.01 was added to every data point to allow zero values to be plotted on the log scale, as indicated in the axis legends. A full correlation analysis to evaluate co-expression patterns of the three genes is shown in the table below.

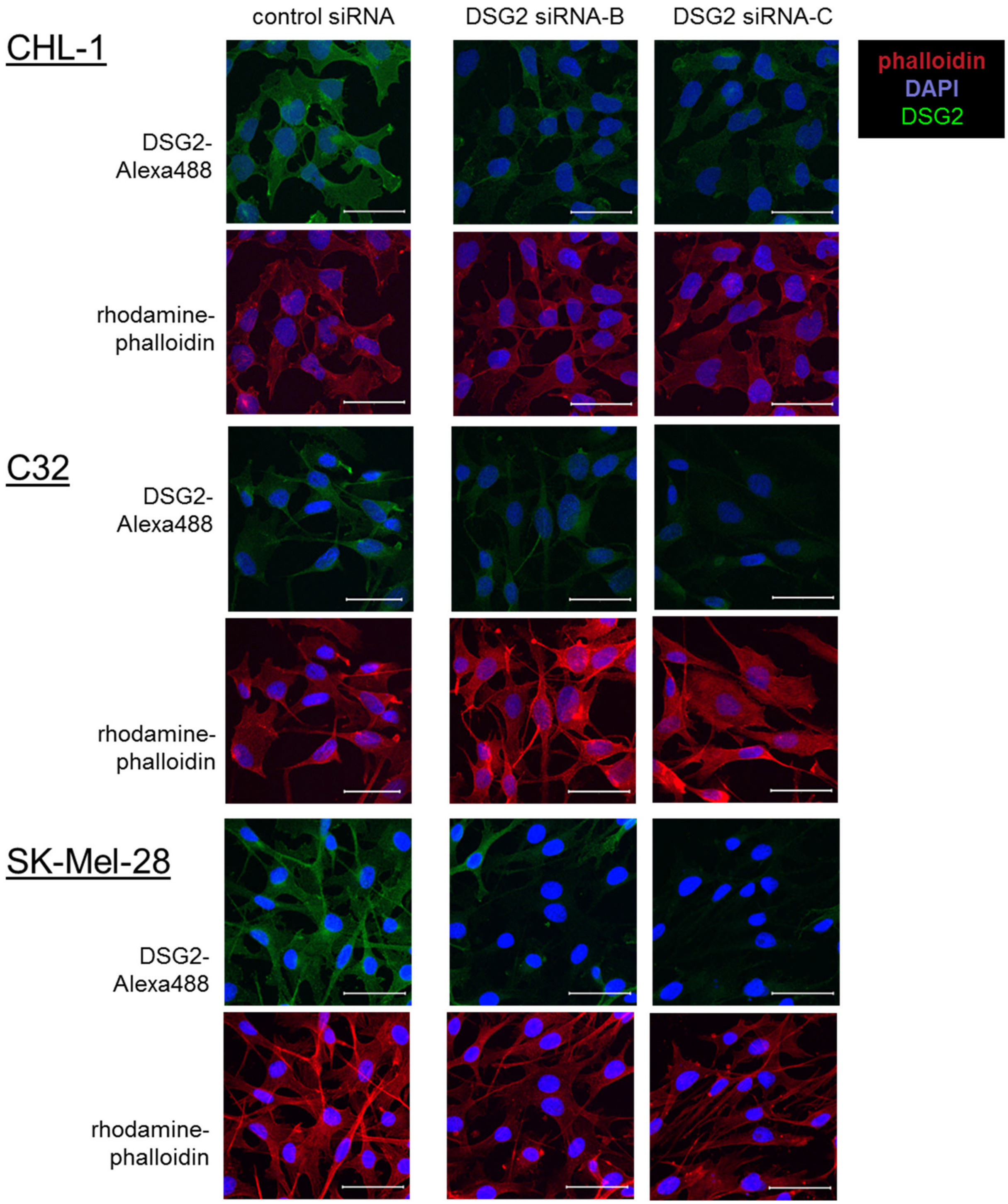

Supplementary Figure S3: Effect of DSG2 knockdown on cell shape and cytoskeletal architecture. Melanoma cells transfected with control or DSG2 siRNA were grown on coverslips for 48hr, stained with DAPI (blue), rhodamine-phalloidin (red) and anti-DSG2 and analyzed by confocal microscopy.

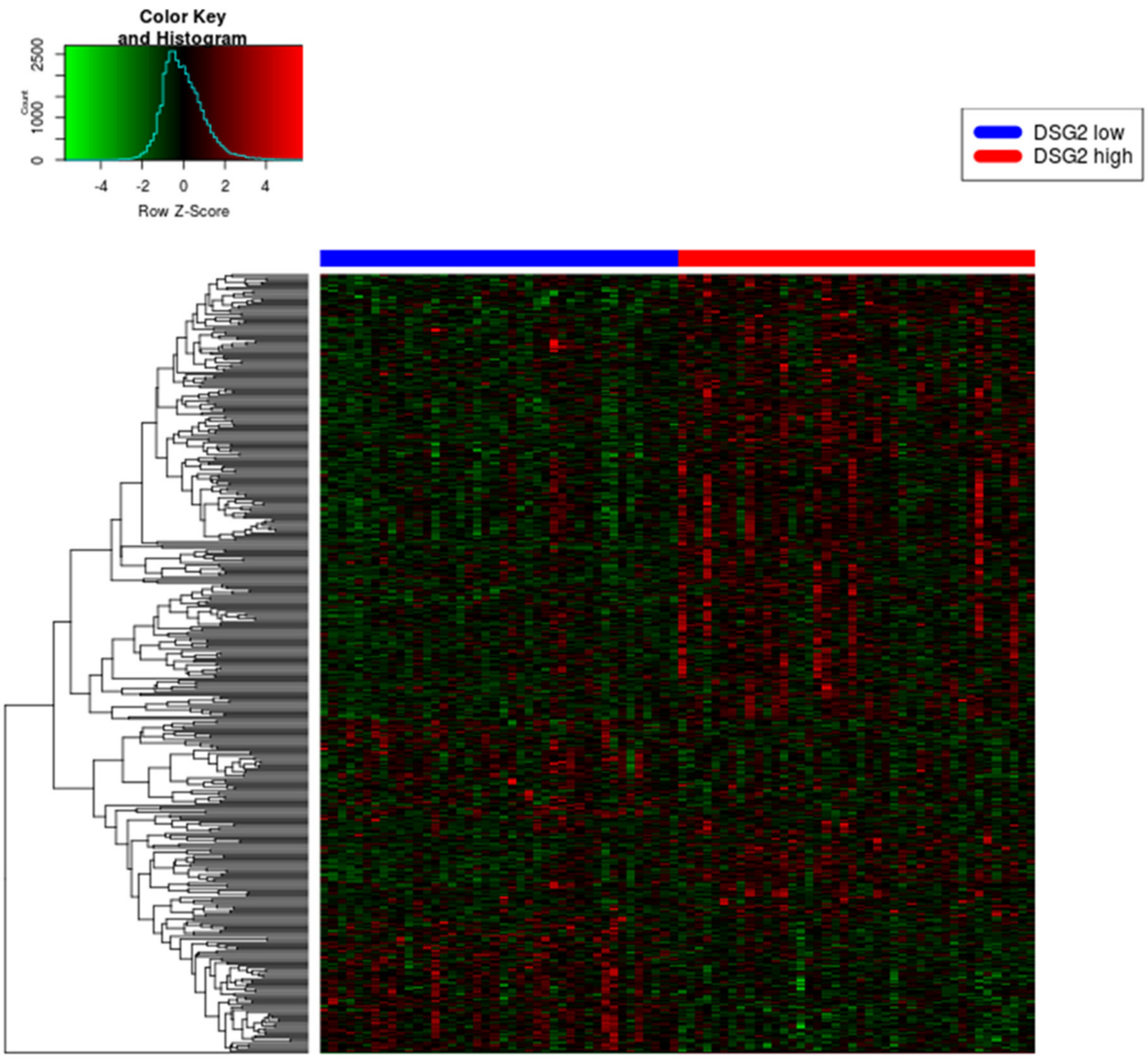

Supplementary Figure S4: Heat map summarizing gene expression differences between DSG2-high and DSG2-low groups within TCGA melanoma samples. TCGA RNA sequencing data was used to identify tumors within the top and bottom 10% of the DSG2 expression range, as shown in Figure 3B. The heat map shows clustering of all genes differentially expressed between the two groups (as detailed in Supplementary Table S1); colors represent distance from the row mean.

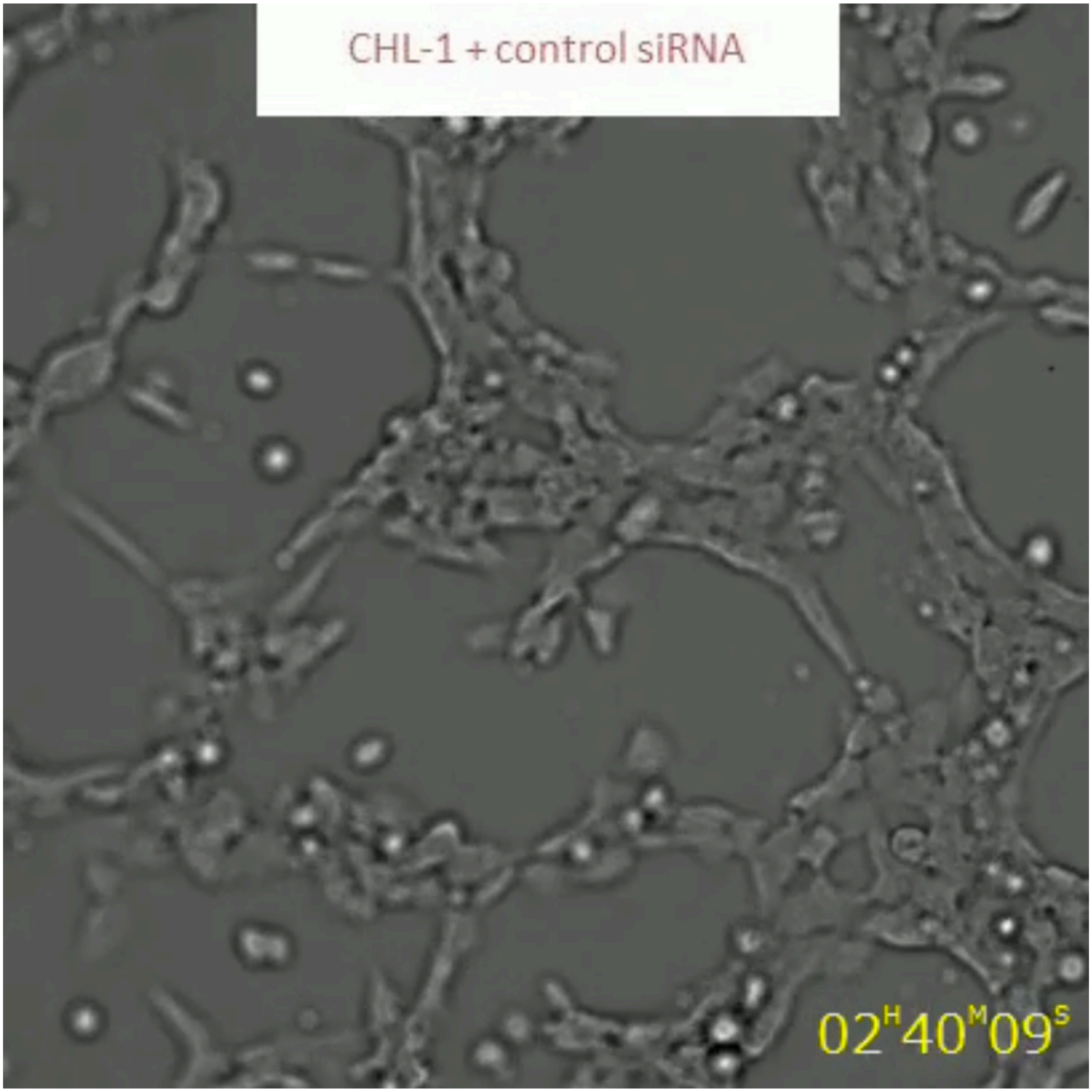

Supplementary Movies S1-S4: Time-lapse imaging of tube formation on Matrigel by CHL-1 or C32 cells. CHL-1 cells (Movies S1-S2) or C32 cells (Movies S3-S4) were transfected with control siRNA (Movies S1 and S3) or DSG2 siRNA (Movies S2 and S4) and 48-72hr later seeded on Matrigel. Time-lapse imaging was initiated within 15 min of seeding, by placing the culture slide in a CV1000 confocal scanner box and capturing images every 5 minutes. Videos were created using the CV1000 integrated software at a frame rate of 5 frames/sec. Note that the Z-plane shifts throughout the video to account for the movement of cells into the Matrigel.

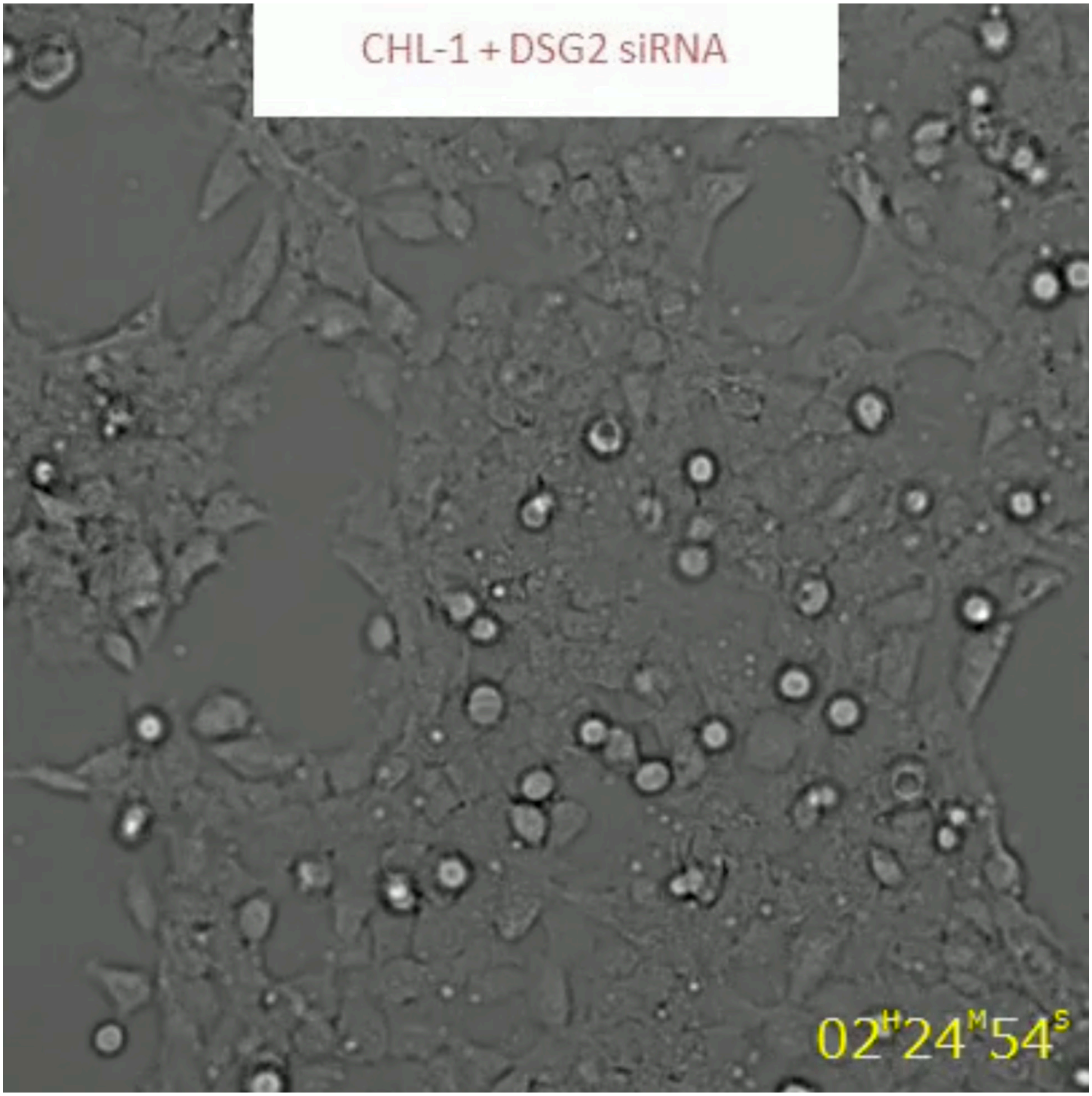

Supplementary Movie S2

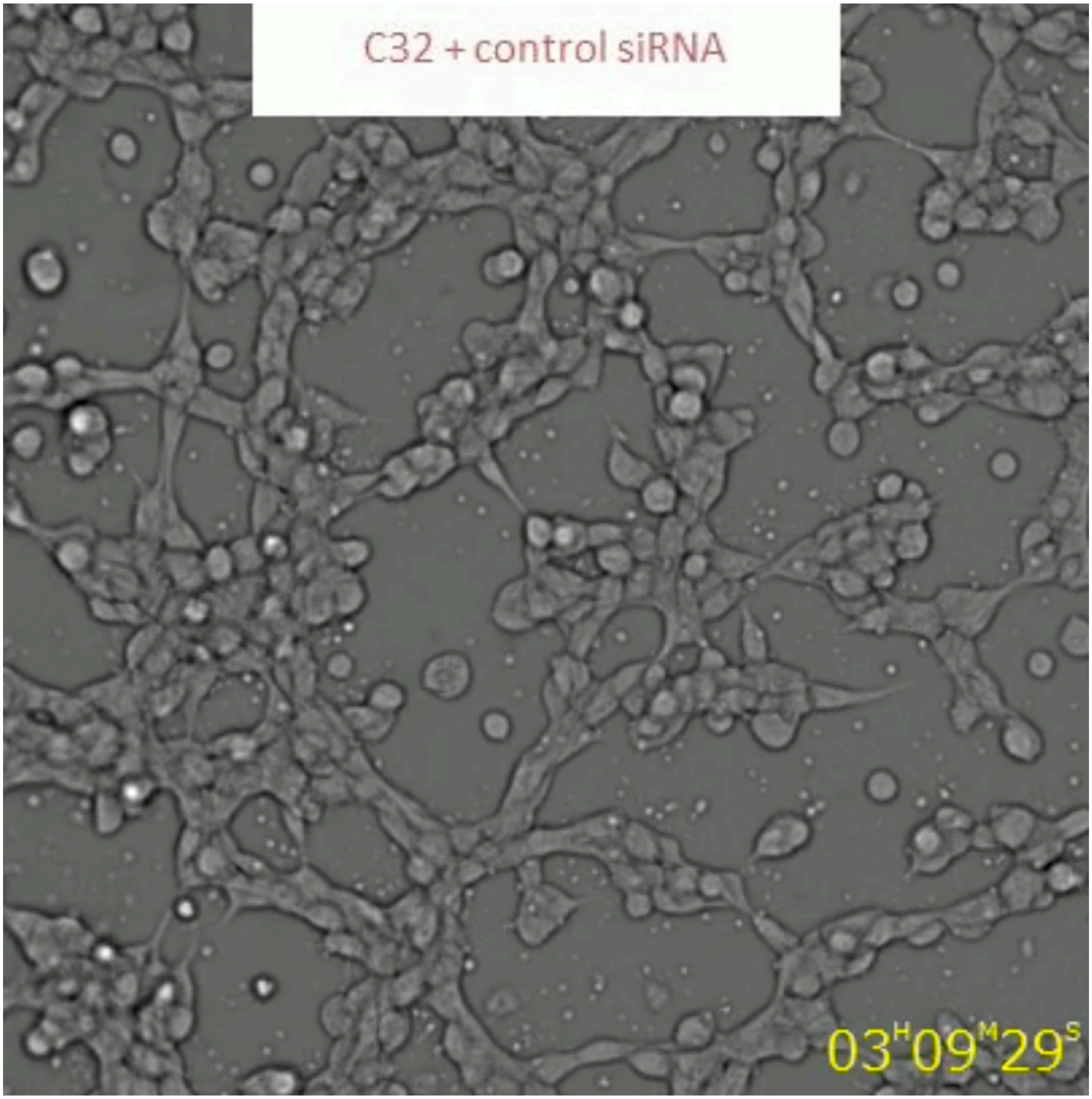

Supplementary Movie S3

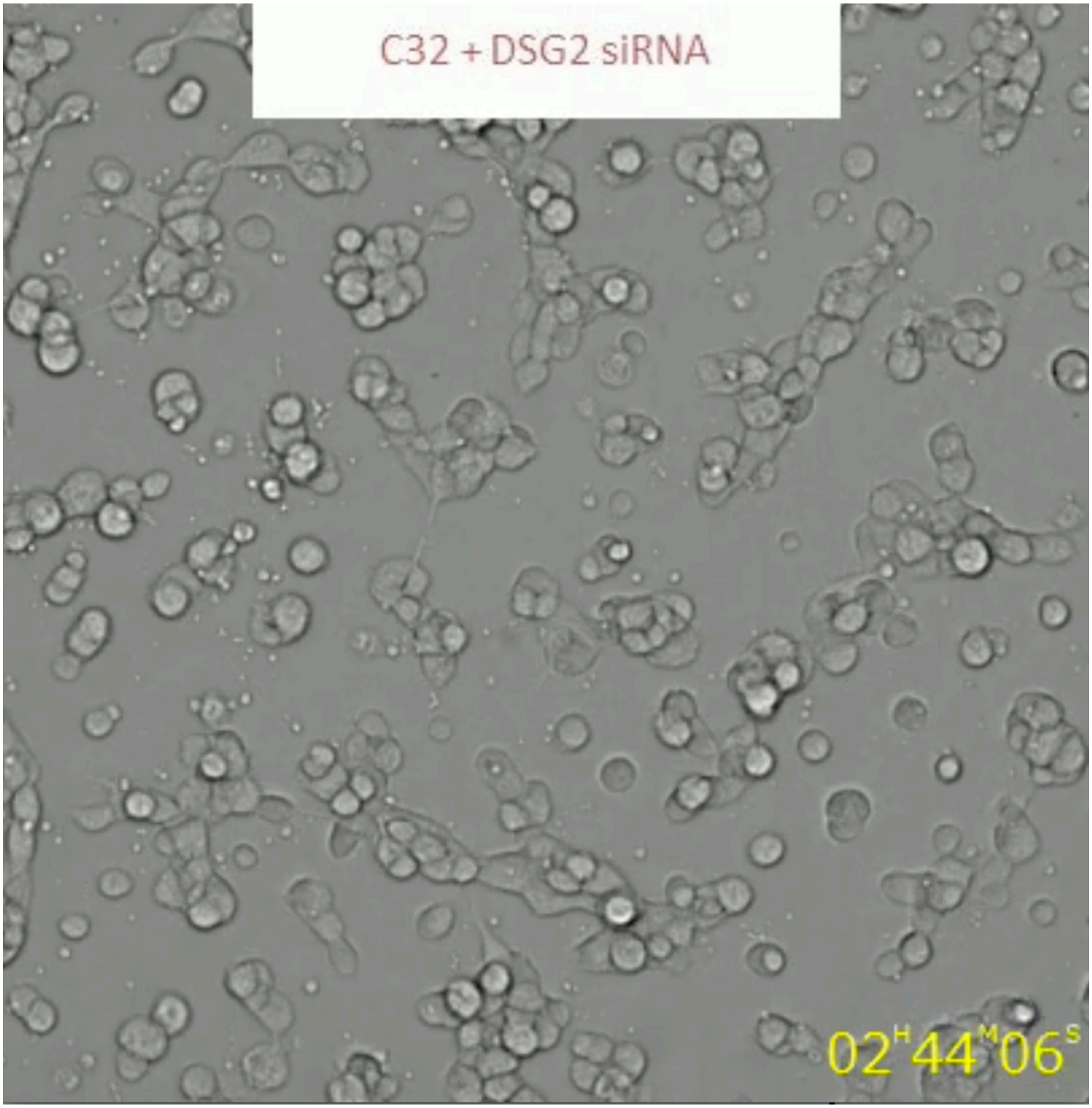

Supplementary Movie S4

Supplementary Table S1: List of genes differentially expressed between the DSG2-high and DSG2-low melanoma subsets within TCGA dataset

See Supplementary File 1
